# Effects of testosterone on contractile properties of sexually dimorphic forelimb muscles in male bullfrogs (*Rana catesbeiana*, Shaw 1802)

**DOI:** 10.1242/bio.20133798

**Published:** 2013-07-25

**Authors:** Aaron R. Kampe, Susan E. Peters

**Affiliations:** Department of Biology, The University of North Carolina at Charlotte, Charlotte, NC 28223, USA

**Keywords:** Forelimb muscles, Sexual dimorphism, Testosterone, Bullfrog

## Abstract

This study examined the effects of testosterone (T) on the contractile properties of two sexually dimorphic forelimb muscles and one non-dimorphic muscle in male bullfrogs (*Rana catesbeiana*, Shaw 1802). The dimorphic muscles in castrated males with testosterone replacement (T_+_) achieved higher forces and lower fatigability than did castrated males without replaced testosterone (T_0_ males), but the magnitude of the differences was low and many of the pair-wise comparisons of each muscle property were not statistically significant. However, when taken as a whole, the means of seven contractile properties varied in the directions expected of masculine values in T_+_ animals in the sexually dimorphic muscles. Moreover, these data, compared with previous data on male and female bullfrogs, show that values for T_+_ males are similar to normal males and are significantly different from females. The T_0_ males tended to be intermediate in character between T_+_ males and females, generally retaining masculine values. This suggests that the exposure of young males to T in their first breeding season produces a masculinizing effect on the sexually dimorphic muscles that is not reversed between breeding seasons when T levels are low. The relatively minor differences in contractile properties between T_+_ and T_0_ males may indicate that as circulating T levels rise during breeding season in normal males, contractile properties can be enhanced rapidly to maximal functional levels for breeding success.

## Introduction

A select group of forelimb muscles in frogs, including the wrist and elbow flexors, are larger in males and also differ from females in a number of physiological properties (e.g. lower fatigability) (Brazier Howell, 1935; Yekta and Blackburn, 1992; Peters and Aulner, 2000; Clark and Peters, 2006; Navas and James, 2007; Ishii and Tsuchiya, 2010). It is thought that this sexual dimorphism results because males use their forelimbs in behaviors that are not typical of females; in grappling with other males and also in clasping females in the mating embrace known as amplexus. Their dimorphic characteristics presumably enhance the males' ability to compete for resources and for females, thereby increasing overall reproductive success, so that we assume these features are under strong selective pressure.

Bullfrogs (*Rana catesbeiana*, Shaw 1802) are useful to study because of their large size and their mating behavior which is typical of many anuran species. They most often mate in large ponds where they set up and defend territories scattered throughout the pond (Emlen, 1976). Males congregate in large groups to compete for mates by calling towards one another and towards females (Wilczynski et al., 2005). The procurement of oviposition sites is the main force driving the organization and distribution of males since females almost always lay their eggs in the male's territory (Howard, 1978). Dominant males who have the largest territories stake claim to the central location of breeding aggregations (Emlen, 1976). Due to this competition, fighting typically ensues which involves grappling between males. They grasp each other belly-to-belly with their flexed forelimbs, abducting their thumbs into each other's sides and pushing or lifting each other in an effort to displace a rival from the disputed territory (Howard, 1978).

Once a female is attracted to a particular male and his territory, the male clasps her in amplexus using a forelimb posture similar to that used in male–male grappling. He flexes his arms around the female, this time from behind, clinging to the “waist” or axillary region. He then abducts his thumbs into her belly and thereby stimulates the female until she releases her eggs (Howard, 1978). Amplexus lasts approximately two hours on average in bullfrogs, but some anuran species maintain this posture for up to a week (Wells, 1977; Howard, 1978). Since amplexus puts the male in position to fertilize the eggs, the ability to perform amplexus effectively would presumably be subject to strong natural selection. Thus, we would expect forelimb muscles in males to have functional properties that would enhance both their grappling ability and their ability to establish and maintain amplexus to ensure their reproductive success.

Previous studies on anuran forelimb muscles document sex differences in mass and energy stores (Yekta and Blackburn, 1992) and in fiber types and sizes (Rubinstein et al., 1983; Dorlöchter et al., 1994). Most recently, several studies have shown that these forelimb muscles in males exhibit significantly larger isometric forces and longer twitch contraction (CT) and half-relaxation (½ RT) times than females (Peters and Aulner, 2000; Clark and Peters, 2006; Navas and James, 2007; Ishii and Tsuchiya, 2010). Moreover, Peters and Aulner discovered that in bullfrogs the prolonged relaxation times allow male muscles to sustain force with little decrease for long periods (>2 sec) between stimuli, resulting in reduced fatigability (Peters and Aulner, 2000). This ability may be a key feature in minimizing energy expenditure during prolonged periods of amplexus. The same results have been documented in the marine toad (*Bufo marinus*) (Clark and Peters, 2006), the European common frog (*Rana temporaria*) (Navas and James, 2007) and the Japanese brown frog (*Rana japonica*) (Ishii and Tsuchiya, 2010), suggesting that slow relaxation and sustained force may be synapomorphic features among anurans (Clark and Peters, 2006).

What triggers the expression of these sexually dimorphic features? Testosterone (T) is an obvious candidate. The most thoroughly studied sexually dimorphic muscles in anurans are those of the larynx (e.g. Kelley, 1997). Testosterone concentration is thought to control the development and maintenance of the dilator muscle of the larynx, which is much larger in males than in females (Coady et al., 2005). Androgen receptors are also known to be more concentrated in the laryngeal muscles in male bullfrogs than in females (Boyd et al., 1999).

A few studies have attempted to test the relationship between T levels and functional properties in the male forelimb muscles of anurans by castration to eliminate the main source of T (Sidor and Blackburn, 1998; Dorlöchter et al., 1994; Regnier and Herrera, 1993). These studies have concentrated on only one dimorphic muscle directly related to amplexus, the flexor carpi radialis (FCR). No studies have been done to correlate the effects of T in other sexually dimorphic forelimb muscles, and none have studied testosterone's effects in the non-dimorphic forelimb muscles. In addition, none of the earlier studies that manipulated T levels directly measured the contractile properties of these muscles in intact animals at naturally occurring muscle lengths, with the nerves and blood vessels maintained.

Investigating how hormones affect the sexually dimorphic musculature involved in territory acquisition, defense and amplexus helps elucidate their importance in such behaviors. We know larger muscles (by mass) in males generate more force (Peters and Aulner, 2000). Testosterone is a main regulator of muscle atrophy and therefore maintains muscle mass, and presumably, muscle force. However, we do not know to what degree T affects other muscle properties such as contraction and relaxation speeds and fatigability. In this study, we examined the effects of T on contractile properties in dimorphic and non-dimorphic forelimb muscles in male bullfrogs to determine whether high levels of circulating T in males are responsible for the sexual dimorphism in multiple contractile properties of these forelimb muscles.

## Materials and Methods

### Myology

**Fig. 1.**
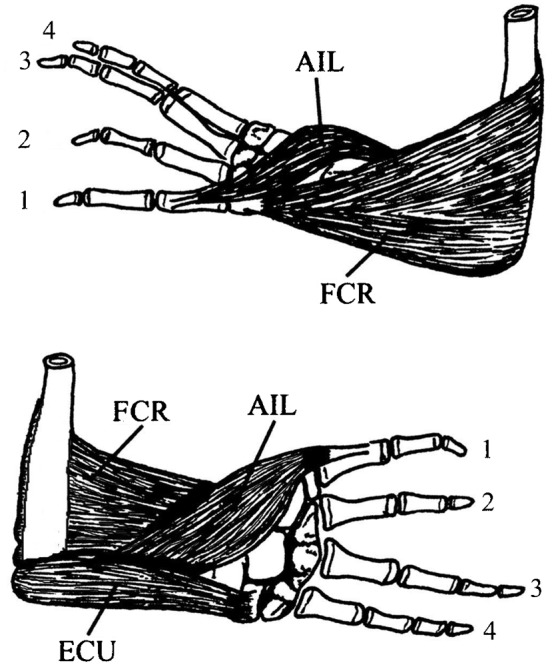
Medial (upper) and dorsolateral (lower) views of the right forelimb of the bullfrog show the muscles under study. Sexually dimorphic muscles include the abductor indicus longus (AIL) and flexor carpi radialis (FCR). AIL originates on the proximal one third of the dorsolateral surface of the radioulna. It inserts medially and distally on the first metacarpal, so that contraction abducts the first digit. FCR is a medial flexor of the wrist, originating along the medial side of the humerus over approximately the distal one third of the bone. It inserts on the medial surface of the radiale. The non-dimorphic extensor carpi ulnaris (ECU) originates laterally at the distal end of the humerus and inserts on the dorsolateral side of the wrist, on the ulnare. Its contraction produces lateral dorsoflexion of the wrist.

The muscles used in this study (Fig. 1) include the abductor indicus longus (AIL) which abducts the first digit. This is the most medial digit and abduction while in the amplexus posture presses its dorsomedial surface into the female's belly, holding her dorsal surface against his belly. Size and contractile properties of AIL were found to be highly sexually dimorphic in bullfrogs (Peters and Aulner, 2000). Another sexually dimorphic muscle is the flexor carpi radialis (FCR) (Fig. 1). This muscle can both flex the elbow and flex the wrist medially so the arms encircle the female during amplexus or a rival male during grappling. A non-sexually dimorphic muscle was studied as a control for general effects of T; this is the extensor carpi ulnaris (ECU) (Fig. 1). It extends the wrist laterally and would have no role in amplexus or grappling. Previous comparison of contractile properties of ECU in bullfrogs found no differences between the sexes (Peters and Aulner, 2000).

### Castration

Adult male bullfrogs were obtained from a supplier (Charles Sullivan, Nashville, TN) and were housed in a vivarium under 12 hr light and 12 hr dark. They were fed a diet of crickets supplemented with feeder fish. The bullfrogs were castrated following procedures outlined by McCreery and Licht under an approved Institutional Animal Use protocol (McCreery and Licht, 1984). They were anesthetized using tricaine methane sulfonate (MS222; 200 mg/kg body mass), injected subcutaneously in the abdominal region. An incision approximately two cm in length was then made in the posterior abdomen, lateral to the midline. This avoids the midventral abdominal vein and both testes were accessed via this single incision. The mesorchium of each testis was ligated using 2–0 suture silk and each testis was removed, leaving the fat bodies intact. Silastic tubing (1 cm long; ID–OD = 1.02–2.16 mm; Dow Corning, Midland, MI) had been prepared in advance; half were empty and half were filled with crystalline testosterone (17β-Hydroxy-3-oxo-4-androstene; Sigma–Aldrich, St. Louis, MO) and sealed with silicon sealant. One tube was placed into the abdominal cavity of each frog. Randomly, half (15) of the bullfrogs received empty implants (T_0_ individuals), while the other half (16) received replacement T implants (T_+_ individuals). Following gonadectomy and implantation, the abdominal muscle and skin were sutured separately. Post-surgery antibiotics were administered (oxytetracycline hydrocholoride, 10–15 µg) once a day for three days. Analgesic (diphenhydramine, 50 mg) was also administered during surgery and thereafter as needed to minimize pain. Following surgery the frogs were held in individual aquaria and, as healing of the incisions allowed (approx. 3–4 days), the animals were exercised at least three days a week by encouraging hopping down a runway. They were also monitored for normal feeding and hopping behavior.

### Contractile measurements

After 8–9 weeks of recovery to allow T levels and contractile properties to stabilize (Regnier and Herrera, 1993), experimental surgery was performed on animals that were pithed following anesthesia with MS222. The two sexually dimorphic muscles (FCR and AIL) and one non-dimorphic muscle (ECU) were tested for their contractile properties in both T_+_ and T_0_ animals. Surgery was done to expose the nerves and muscles. Blood vessels were carefully maintained, and the nerves and muscles were frequently bathed using amphibian Ringers.

FCR is innervated by the radial nerve and both AIL and ECU are innervated by the ulnar nerve. Because the individual muscle nerve branches could not be accessed, we had to stimulate the entire ulnar nerve to test either AIL or ECU. As a result, we could not use the same animals for testing all three muscles. ECU was tested in separate subsets of T_0_ and T_+_ animals. In order to minimize the numbers of animals used, AIL and FCR were tested in the same individuals among subsets of T_+_ and T_0_ animals.

Once the surgical dissection of the muscle under study was complete, a marker tie was made in the insertion tendon (using 2–0 braided suture silk; CP Medical; MedRep Express, Prescott, AZ) as close to the junction of muscle fibers and tendon as possible. A second tie was placed near the muscle origin in connective tissue which did not move as the muscle length was changed. The wrist and elbow were positioned at joint angles replicating standing position, maximum flexion and extension during normal locomotion, and amplexus (known from Peters and Aulner, 2000), and the muscle lengths were measured between the marker ties. Each muscle was then cut free of the bones at the insertion and attached via low-compliance surgical silk (size 0; CP Medical; MedRep Express, Prescott, AZ) to an isometric strain gauge (Grass FT10; Grass Technologies, Astro-med, Inc., West Warwick, RI).

Fine wire stainless steel electrodes (0.08 mm diameter; California Fine Wire, Grover Beach, CA) were implanted into the belly of the muscle to monitor electromyograms (EMG). With circulation intact, muscles were stimulated (Grass S88 square wave stimulator) via their nerves using bipolar stainless steel electrodes. Supramaximal stimulation was produced by determining twitch threshold voltage and then increasing voltage until no further increase in twitch force could be obtained (approx. 2–2.5 times threshold).

The strain gauge was mounted on a rack and pinion so that the attached muscle could be lengthened or shortened. Muscles were stimulated at 2 mm intervals covering the normal range of lengths, and all subsequent tests were performed at the length where maximum isometric tetanic tension occurred. We measured contractile properties commonly found in such studies and which were used in the comparison of male and female bullfrogs by Peters and Aulner (Peters and Aulner, 2000). These included maximum tetanic force (TT) (670 msec train of 0.01 msec impulses at 80 pps) and twitch force (Tw) (single impulse, 0.01 msec duration), as well as twitch contraction (CT) and half relaxation times (½ RT), the force-frequency relationship and fatigability (Peters and Aulner, 2000). We also measured the maximal rate of tension rise (dp/dt) for the twitch. Contraction time was measured from the stimulus artifact to the peak of twitch tension. Half-relaxation time was measured from the peak of twitch force to the point where twitch tension fell to half of its peak value. The rate of force increase in the twitch (dp/dt) was measured from the slope of the linear portion of the rising force trace. In the force-frequency test, the muscles were stimulated for 670 msec (0.01 msec duration) at 5-pulse intervals from 5 to 40 pps, and at 60 and 80 pps. Muscles were rested for one minute between each stimulation train. The fatigability of the muscle was determined over a four minute time period using a 200 msec train of stimuli at 30 pps, once every two seconds (Peters and Aulner, 2000). A fatigue index (FI) (Burke et al., 1971) was calculated as the sum of total force produced during the first 2 min divided by the total force produced over the entire 4 min test taken as a percentage. Thus, an FI of 50 indicates that, during the first half of the test, the muscle produced half of the total force, hence, no fatigue. A fatigue index above 50 indicates increasing fatigue (Peters and Aulner, 2000).

Each experimental muscle, as well as the one from the non-experimental limb, was also harvested from the individual frogs, and their masses were averaged to get individual muscle masses. An estimate of the average cross-sectional area of each muscle was made by dividing the muscle mass (∼volume) by its standing length. We could then calculate the tetanic force per muscle cross-sectional area (kg/cm^2^) for each muscle.

### Enzyme linked immunosorbent assay

Directly after the muscle tests were completed, blood samples (approximately 1 ml) from each individual were taken from the heart. The serum was then isolated (spun at 6000 *g* for 10 min @ 4°C) and stored at −80°C. Testosterone levels were measured using an enzyme linked immunosorbent assay (ELISA) (Testosterone EIA kit; Enzo Life Sciences, Farmingdale, NY) to make sure that implanted frogs had circulating levels in the normal range and untreated frogs had low levels. The sensitivity of the kit is 5.67 pg/mL. A standard curve was made using data from the premeasured testosterone standards provided. The serum and standard samples underwent the antibody reaction and optical densities were measured at 405 nm on a spectrophotometer (Multiscan GO; Thermo Fisher Scientific, Waltham, MA). Testosterone levels from the experimental frogs were then determined from the standard curve.

### Data analysis

Since muscle size varies with body mass, values for force production in each muscle (Tw and TT) as well as muscle mass and cross-sectional area were compared in one-way ANCOVAs using body mass as a covariate to analyze the differences between the T_+_ and T_0_ individuals. For each muscle, the rate of force increase in the twitch (dp/dt) was compared between T_+_ and T_0_ animals in an ANCOVA using their maximum twitch force as the covariate. One-way ANOVAs were used for all size-independent contractile data including CTs, ½ RTs, TT/cm^2^ of muscle cross-section and fatigue indices. All comparisons were subjected to the sequential Bonferroni adjustment for multiple comparisons. Six parameters (TT, Tw, CT, ½ RT, muscle mass, FI) along with the percent of sustained force (see Results) were compared in a binomial test to determine whether data for T_+_ individuals, taken as a whole, varied from the T_0_ individuals in the direction expected for masculinized properties. This test calculates whether the pattern of means across all parameters is randomly distributed or whether they are skewed toward a theoretically expected distribution of observations, in this case toward the expected masculinized direction for each property (known from previous data: Regnier and Herrera, 1993; Peters and Aulner, 2000).

One way ANOVA was used to compare the T levels measured in the ELISA test for T_+_ and T_0_ individuals. Force-frequency and sustained contraction data were calculated at each appropriate interval in percent of maximum and compared between T_+_ and T_0_ individuals for each muscle using one-way ANOVA as well.

Since the means, standard errors, and sample sizes for contractile properties of AIL and FCR in normal male and female bullfrogs were known from a previous study (Peters and Aulner, 2000) we were able to compare these to the T_+_ and T_0_ males using one-way ANOVAs. Tukey's tests were then used *post hoc* to analyze the pairwise differences between each group for each property.

We have noted throughout our results the comparisons in which *P* values fall between 0.05–0.1. Though we cannot claim that these are statistically significant differences, they may indicate trends that have biological significance, e.g. a slight slowing of relaxation in muscles of breeding males (high T) compared to non-breeding (low T) may enhance clinging ability (see Discussion of sustained force).

## Results

All data described in this study were collected from both T_+_ and T_0_ individuals ranging in size from 150–332 g (grand mean±s.d. = 236.5±56.8 g; N = 26). There was no significant difference in the mean body masses of the T_+_ (mean = 224.1±45.9 g; N = 13) and T_0_ (mean = 248.9±65.4 g; N = 13) frogs used in any of the comparisons.

### Efficacy of castration and replacement

Testosterone levels in the T_+_ and T_0_ individuals were determined using an ELISA test to monitor the efficacy of implantation and make sure T levels were within previous known ranges of wild-caught bullfrogs (Murphy et al., 2006). Testosterone levels in bullfrogs vary throughout the year (Murphy et al., 2006); non-breeding frogs may have nearly undetectable levels of T while breeding individuals are much higher. Thus, it was important to measure the difference in T concentrations between T_+_ and T_0_ individuals. Indeed the T_+_ frogs had T levels (mean±s.e.m. = 9.7±2.3 ng/ml) which fell within the range of normal breeding males (mean±s.e.m. in May: 8.2±0.08 ng/ml) (Licht et al., 1983), and our T_0_ individuals were significantly lower (mean±s.e.m. = 0.46±0.09 ng/ml; *P* = 0.002) than normal and T_+_ individuals.

### Contractile properties

The mean values ± s.e.m. for all contractile properties for each muscle are shown in Table 1. None of the comparisons between T_+_ and T_0_ individuals differed significantly in the non-dimorphic ECU. Among the two sexually dimorphic muscles, a pair-wise comparison found that muscle mass was not significantly greater in the T_+_ individuals for either dimorphic muscle, though the difference in muscle mass for FCR approached significance (*P* = 0.07) (Table 1). Maximum twitch force was significantly larger in T_+_ than T_0_ males for the FCR, but the larger Tw in T_+_ males of AIL only approached significance (*P* = 0.1). Maximum tetanus was significantly larger in the AIL of T_+_ individuals but values did not reach significance in the FCR (*P* = 0.1). The larger maximum tetanic force in T_+_ AILs also resulted in greater tetanic force/muscle cross section than in the T_0_ AILs (Table 1). However, the tendency of the T_+_ FCR to be somewhat larger in cross section than in T_0_ males (*P* = 0.06) resulted in no significant difference in the tetanic force/muscle cross section. Differences between T_+_ and T_0_ males for CT and ½ RT also did not reach significance in any of the muscles. A closer examination shows that in T_+_ FCRs, both CT and ½ RT tended to slow relative to the T_0_ muscles (*P* = 0.08 and *P* = 0.1, respectively; Table 1). In addition, the ½RTs in ECU were significantly shorter than the CTs in both T_+_ and T_0_ males (*P* = 0.01 and *P* = 0.002, respectively), but the CTs and ½ RTs are approximately equal in AIL and FCR in both T_+_ and T_0_ males. Thus, the dimorphic muscles have relatively slower ½ RTs.

**Table 1.**
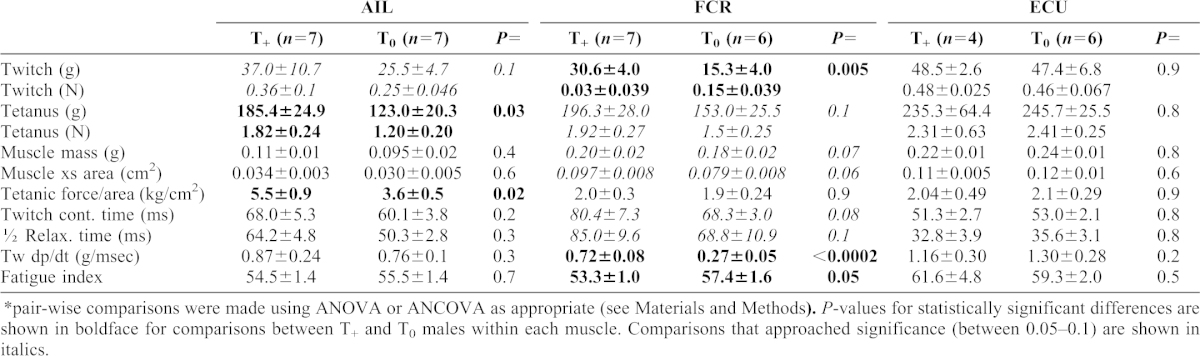
Comparison of muscle sizes and contractile properties (mean ± s.e.m.).* *pair-wise comparisons were made using ANOVA or ANCOVA as appropriate (see Materials and Methods). *P*-values for statistically significant differences are shown in boldface for comparisons between T_+_ and T_0_ males within each muscle. Comparisons that approached significance (between 0.05–0.1) are shown in italics.

The rate of force development during the rising phase of the twitch (dp/dt) was compared for each muscle between the T_+_ and T_0_ males (Table 1). This value did not differ significantly among the AILs or ECUs, but the T_+_ FCRs did increase highly significantly faster during the twitch than in the T_0_ males (*P*<0.0002). A final comparison in which FCR differed significantly was the fatigue index. FCR in T_+_ individuals was significantly lower than in T_0_ individuals, suggesting greater fatigue resistance with higher T levels. These indices did not differ between T_+_ and T_0_ individuals in either the AIL or ECU.

Thus, the T_+_ and T_0_ males differed significantly (*P*<0.05 or less) in only two parameters in AIL (maximum tetanic force and tetanic force/muscle cross section) with one other approaching significance (twitch force) (*P* = 0.1). In FCR, three parameters (maximum twitch force, dp/dt, and FI) differed significantly, but five others approached significance with *P* values between 0.05–0.1. None of the differences in the ECU males reached or approached statistical significance.

### Sustained force

**Fig. 2.**
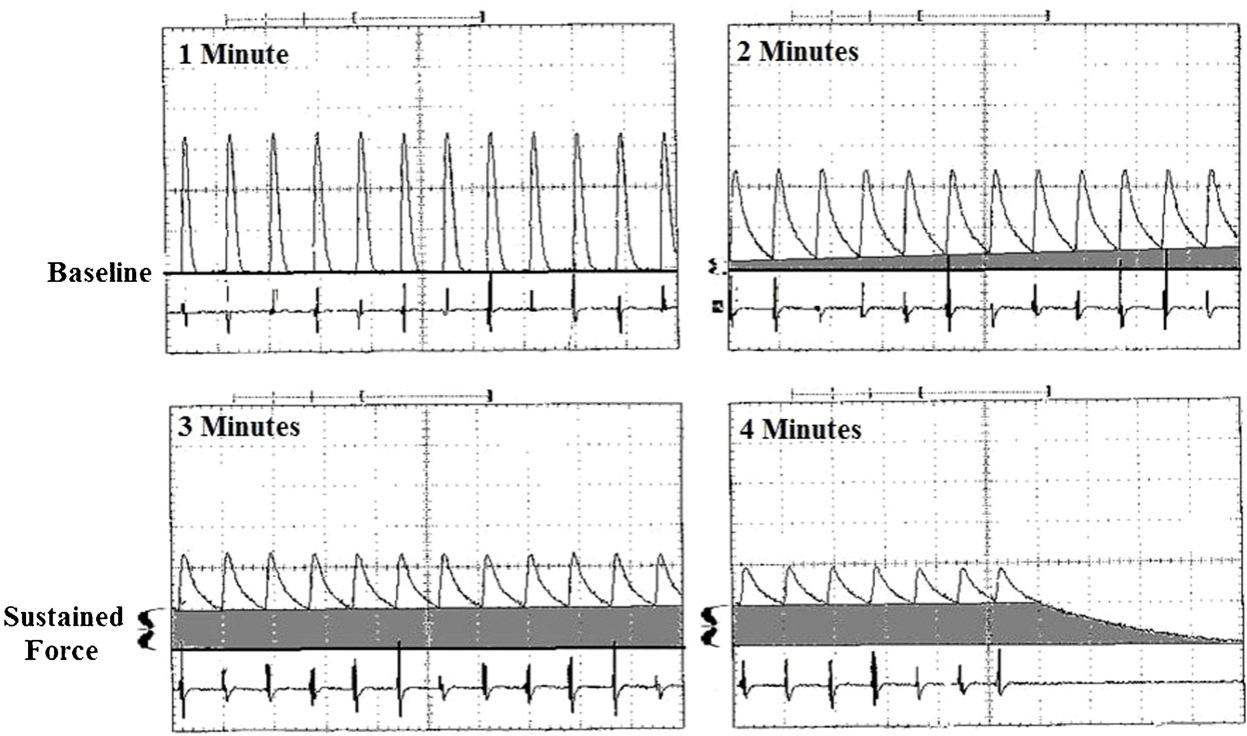
Sustained force development during the fatigue test is shown for a representative T_+_ AIL. The upper line on each panel displays the force produced during intermittent stimulus trains (200 msec duration, every 2 sec @ 30 pps) and the EMG trace is shown below. During the first minute, the brief tetanic bursts rise and fall back to baseline. By 2 min, the relaxation times have elongated and the force trace fails to return to baseline during the 2 sec between stimulus trains. The shaded area shows the amount of unrelaxed force (sustained force) as it increases throughout 3 min and 4 min. By the end of the test over half of the total force is comprised of sustained force, and when the stimulus trains cease it takes nearly 10 seconds for the muscle to relax back to the original baseline (sweep speed = 2.5 sec/division).

The four minute fatigue test used to calculate the fatigue index is also used to examine the phenomenon known as sustained force (Peters and Aulner, 2000). Sustained force is the amount of unrelaxed force that develops over the four minute test when muscles are stimulated intermittently (see Materials and Methods). Fig. 2 illustrates this phenomenon. Initially during the fatigue test force rises and falls sharply during brief, intermittent tetanic stimulations. However, over four minutes, unrelaxed force develops due to an extreme elongation of relaxation time so that the muscle does not relax to baseline during the two second intervals between stimulus trains (Fig. 2). Sustained force was never observed in either T_+_ or T_0_ individuals for ECU, but it occurs in T_+_ and T_0_ individuals in both of the sexually dimorphic muscles.

**Fig. 3.**
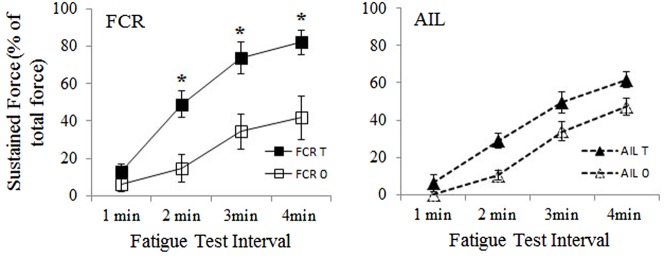
For both T_+_ (T) and T_0_ (0) individuals, sustained force in AIL and FCR is expressed as a percent of the total force generated at each one-minute interval during the four-minute fatigue test. Asterisks indicate significant differences at the indicated time intervals.

Fig. 3 shows the percent of total force comprised of sustained force for T_+_ and T_0_ males compared at 1, 2, 3, and 4 minutes for both AIL and FCR. In AIL, sustained force reached 50–60% of total force by four minutes. Differences between the T_+_ and T_0_ individuals in the amount of sustained force during the fatigue test did not reach significance at three and four minutes (*P* = 0.08 and *P* = 0.07, respectively) and, allowing for multiple comparisons, only approached significance at two minutes (*P* = 0.02). So the magnitude of difference in AIL was low. In the T_+_ males, FCR reached significantly higher levels of sustained force than in T_0_ individuals at two minutes into the fatigue test (mean = 49.0±7.1% vs 14.7±7.4%; *P* = 0.010) and also at three minutes (mean = 73.7±8.4% vs 34.4±9.4%; *P* = 0.014). By four minutes the sustained force in the T_+_ FCRs reached a much higher percent of total force than in the T_0_ FCRs (mean = 82.1±6.4% vs mean = 41.8±11.8%; *P* = 0.017).

### Trends in the data

In both of the sexually dimorphic muscles the differences that were statistically significant between T_+_ and T_0_ males were as expected in light of the assumed role of T in masculinizing the muscles. However the majority of comparisons between the sexually dimorphic muscles did not reach significance, partly due to the relatively high variation found in the data and the statistical adjustment for multiple comparisons. Though few of the pair-wise comparisons among contractile properties differed significantly between the T_+_ and T_0_ males, the non-significant differences in the means all trended in the directions expected if T masculinizes the contractile properties and lack of T results in decreased masculine effects. For example, normal male properties (Peters and Aulner, 2000) that are consistent with the T_+_ males include maximum Tw and TT forces as well as muscle masses tending to be larger in T_+_ animals, CTs and ½ RTs being longer, the FI being lower (Table 1), and the amount of sustained force during the fatigue test tending to be greater in T_+_ males (Fig. 3). The binomial test showed that these trends in the data taken as a whole were significantly different between T_+_ and T_0_ males for the sexually dimorphic muscles (*P* = 0.008) but not for the non-dimorphic one. This analysis suggests that T has masculinizing effects in the sexually dimorphic muscles; however, the magnitude of the variation in our samples limited the power to define differences when multiple properties were analyzed in pair-wise comparisons.

**Fig. 4.**
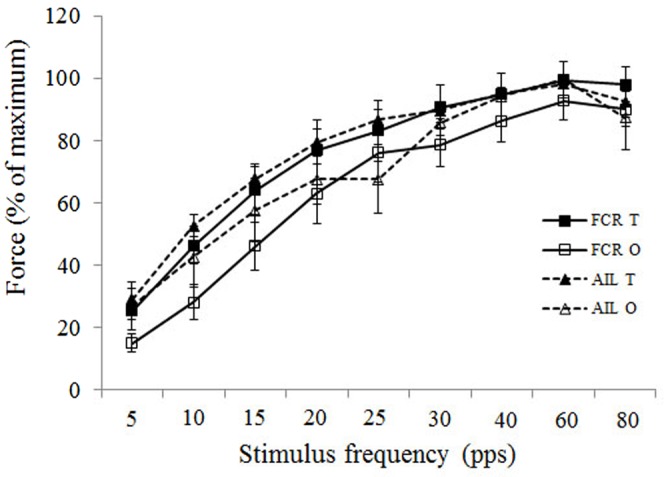
Force/frequency curves compare the amount of force (as a percentage of maximal force) in T_+_ and T_0_ muscles at stimulus frequencies varying between 5 and 80 pulses per second. No significant differences between T_+_ and T_0_ males were found in either muscle at varying frequencies.

Changes in maximum tetanic force at increasing frequencies (force-frequency) for the two dimorphic muscles are shown in Fig. 4. There were no significant differences in the force produced at any of the stimulus frequencies when each muscle was compared between T_+_ and T_0_ individuals. Again, variation in the data limited our ability to discern significant differences in the pairwise comparisons at each frequency, but the T_+_ means tended to be a consistently higher percent of maximum tetanic force than the T_0_ means throughout the test, approaching significance for AIL at 25 pps (*P* = 0.06) and for FCR at 10 pps (*P* = 0.08), 15 pps (*P* = 0.08), 30 pps (*P* = 0.07), 40 pps (*P* = 0.07), and 60 pps (*P* = 0.08).

So overall, more of the FCR properties were significantly different or approached significance (*P* = 0.05–0.1) when the T_+_ and T_0_ males were compared (Table 1; Fig. 3). Fewer AIL comparisons reached or approached significance, though masculine trends were apparent (significantly larger TT and greater TT/muscle xs; trending toward greater Tw and sustained force). None of these properties approached significant differences in the ECU.

### Comparison with male and female properties

**Fig. 5.**
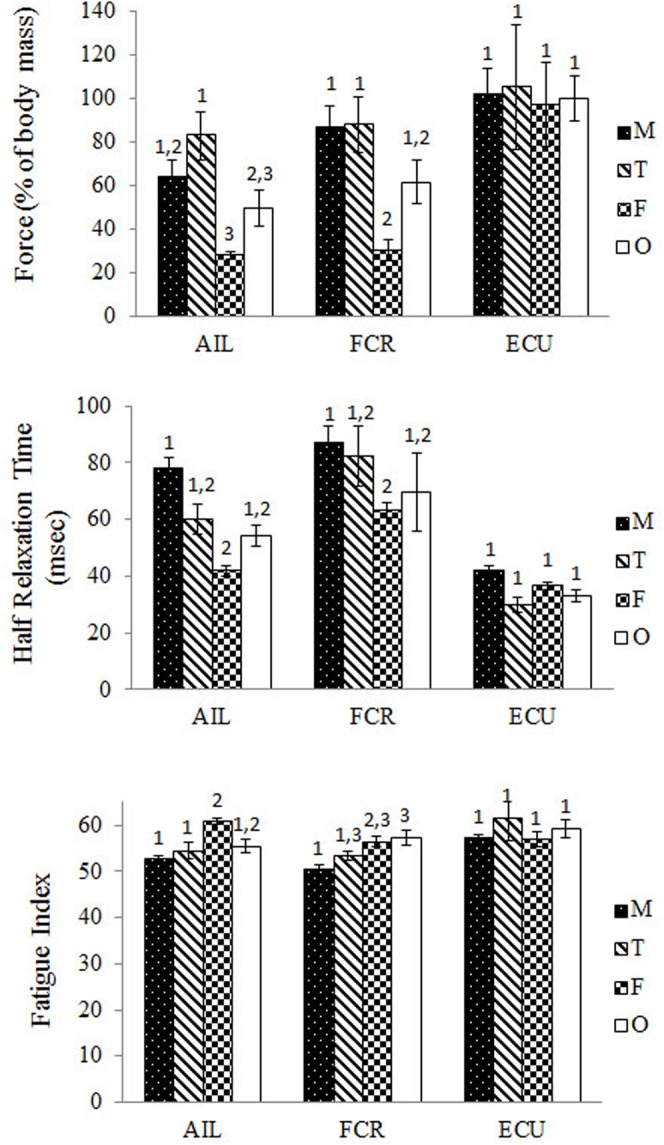
Comparison of normal males and females (data from Peters and Aulner, 2000) with our data for the T_+_ and T_0_ males. Values that were compared include: maximum tetanic force scaled to body mass among all groups (top), half relaxation times in msec (middle), and the fatigue index (bottom) among all groups. Groups sharing the same number are not significantly different from each other. M = normal males; F = females; T = T_+_ males; 0 = T_0_ male.

Previous data on normal, untreated male and female bullfrogs (Peters and Aulner, 2000) were compared to the T_+_ and T_0_ males (Fig. 5). In the AIL, the T_+_ group produced the highest tetanic force and was significantly greater than that of both T_0_ males (*P* = 0.009) and females (*P* = 0.01) (Fig. 5). However, there was not a significant difference between normal males and T_+_ males or between T_0_ males and females. For the FCR, the mean tetanic force did not differ among normal, T_+_ or T_0_ males. There was a significant difference between both normal and T_+_ males and females (*P* = 0.003; *P* = 0.002, respectively), but no difference between T_0_ males and females. For the ECU, there was no difference in tetanic force among any group.

With regard to half relaxation times (½ RT) a similar pattern exists among means but with fewer differences (Fig. 5). For both dimorphic muscles the only significant difference was between normal males and females (for AIL, *P* = 0.007; for FCR, *P* = 0.009). There were no differences in relaxation times between normal, T_+_ and T_0_ males. Neither T_+_ nor T_0_ males differed significantly from the females. Contraction times (CT) show the same pattern of similarities and differences as did the ½ RT. No mean differences were found in ECU among the groups for either CT or ½ RT.

The fatigue indices (Fig. 5) show that in AIL the normal and T_+_ males had the least fatigue and were significantly different from the females (*P*<0.001; *P* = 0.006, respectively) which had the highest index (most fatigue). The T_0_ males were intermediate in being not significantly different from either of the other males or from the females. For FCR, the T_+_ males and normal males did not differ, but the normal males were significantly different from the T_0_ males which were more fatigable (*P* = 0.008). The T_0_ FIs were also the same as the females, so in this instance, the females were intermediate between the T_0_ males and the normal and T_+_ males. None of the mean FI values for ECU differed among the groups.

In general, then, T_+_ and normal males produced the greatest force and speed values, and lowest fatigue. T_0_ males were typically intermediate between females and the other males in the sexually dimorphic muscles among the properties tested.

## Discussion

Testosterone is responsible for large muscle mass and other masculine properties of vertebrate skeletal muscles (Regnier and Herrera, 1993; Dorlöchter et al., 1994; Catz et al., 1995; Sidor and Blackburn, 1998; Boyd et al., 1999; Coady et al., 2005). These effects of T commonly result in sexual dimorphism. Our aim was to test the extent to which T levels contribute to the sexual dimorphism in forelimb contractile properties in male bullfrogs. This study is important in light of the sexual dimorphism that is limited to the joint flexors of the forelimbs in frogs, suggesting that this dimorphism evolved as an adaptive feature for breeding behaviors (grappling and amplexus), rather than being a global and incidental effect of high T in males.

Data from this study show a mixture of some properties that are more strongly affected by T levels and some that are not. Our results suggest that some dimorphic muscles (FCR in this study) may be more strongly influenced by T levels than others (e.g. AIL). They also show the selective nature of the dimorphism in muscle properties because none of the properties of the non-sexually dimorphic ECU were affected by differences in testosterone. It is important to note that differences between T_+_ and T_0_ males reflect the condition of the muscles 8–9 weeks following castration. Analysis of the T levels showed that the T_+_ males maintained breeding season levels of T, and that the males without T replacement had negligible levels. We assume that T falls rapidly following castration (Ingberg et al., 2012), and we used the 8–9 week recovery period because it had been shown to be effective in stabilizing differences in muscle properties in earlier studies: Sidor and Blackburn found that T replacement maintained the large mass of sexually dimorphic musculature in *Rana pipiens* (Sidor and Blackburn, 1998); furthermore, Regnier and Herrera showed that T replacement resulted in both greater tetanic force and longer ½ RT in the FCR of *Xenopus laevis* (Regnier and Herrera, 1993). Thus, our data may reflect the full extent of differences in properties between T_+_ and T_0_ bullfrogs, but a longer period of low T may have produced more significant differences between the T_+_ and T_0_ bullfrogs.

### Force development

We used two indicators to examine differences in force development among the T_+_ and T_0_ males: force/frequency measurements and dp/dt. Differences among muscles in force production at varying frequencies reflect the dynamics of muscle fiber activation. Although the natural range of frequency input in bullfrogs is unknown, in normal behaviors it is likely to be lower than the 80 pps we used for the supramaximal stimulation. Peters and Aulner found that male AIL muscles produced significantly more force at these lower frequencies (<40 pps) than did female AIL muscles (Peters and Aulner, 2000). They hypothesized that this would be advantageous because at any given frequency in the low range, males could recruit a relatively greater percentage of their maximum force. Thus, at lower frequencies, males would get more out of their muscles for a given neural input. Presumably, minimizing the firing rate of the motor neurons would save energy in males that have to use their forelimbs for prolonged periods in grappling and amplexus.

The percent of maximum force produced by T_+_ and T_0_ males at increasing frequencies in both AIL and FCR was similar within muscle comparisons (Fig. 4). The difference between T_+_ and T_0_ males tended to be greatest in FCR at low frequencies, but differences only approached statistical significance (P between 0.05–0.1 at 10, 15, 30, 40 and 60 pps). AIL did not differ in the percent of maximum force produced at any frequency between T_+_ and T_0_ males. So the lack of testosterone did not greatly change the magnitude of the muscles' response to activation. It is notable that the variation among individuals was highest at the lower frequencies, perhaps signaling that this is a plastic feature and varies widely according to individual and physiological conditions.

The rate of force increase during a twitch did not differ significantly between the T_+_ and T_0_ AIL muscles. However, this difference was the largest of all the comparisons for FCR (T_+_ males generated over 2.5 times the force/msec of the T_0_ males in the linear phase of force increase). This indicates that FCR can generate its force much more rapidly in a single twitch when the muscle is exposed to normal testosterone levels, and that this activation scales back during the non-breeding season. At first, this seems counter intuitive given the extremely long CTs found in the T_+_ FCRs. However, the maximum twitch forces reached in the T_+_ males for FCR are twice as great as in the T_0_ males. This may not account for all of the difference in rate of force increase when compared to the differences in CT. We looked at the FCR twitch traces and found that the linear portions of the trace were a significantly shorter part of the overall time to reach peak force (CT) in the T_+_ males (23.7±2.0 msec = 29.5% of total CT) than in the T_0_ males (33.3±2.1 msec = 48.8% of total CT; *P* = 0.008). So the early linear force increase in T_+_ FCRs is very rapid, but it also slows rapidly before reaching peak twitch force, i.e. produces a more broadly curved trace than in the T_0_ males. This interesting result is consistent with the results from the force/frequency and the sustained force data. In a low frequency tetanic stimulation, the rapid slowing of force development in the T_+_ males would delay the force from entering the falling phase of the trace before the next stimulus in a train occurs, thus pushing the force higher in a stair-step fashion. So at low to moderate stimulus frequencies, we see unfused tetani showing a treppe pattern in which the force builds from one stimulus to the next, resulting in greater maximum force with low frequency stimulation. With intermittent stimulation (as in the sustained force tests – see below), prolongation of relaxation that is most pronounced in T_+_ males leads to a pattern of elevated force but over longer time and with longer breaks between stimulus trains.

Future studies are needed to examine the diversity of myosin isoforms in T_+_ and T_0_ males which may shed light on whether T affects the myosin ATPase gene expression. Other effects, such as Ca^2+^ build up in the cytoplasm (see below), ATP and CPK metabolism should also be examined.

### Sustained force

Peters and Aulner first described the phenomenon of sustained force in sexually dimorphic forelimb muscles of bullfrogs (Peters and Aulner, 2000). Subsequently, the same pattern was found in other anurans (Clark and Peters, 2006; Navas and James, 2007). The physiological basis for this remains unclear, however, it is likely that changes in the sequestration of Ca^2+^ during prolonged and/or intermittent stimulation is involved. Testosterone has been shown to affect SERCA (sarco-endoplasmic reticulum calcium ATPase) activity *in vivo*. Liu et al. found that T inhibits SR Ca^2+^-ATPase pumping activity in a fast twitch muscle in mice (Liu et al., 2008). In this way a higher concentration of Ca^2+^ binds troponin C and slows muscle relaxation overall. If, during prolonged contraction in a high-T environment, the kinetics of the SR Ca^2+^-ATPase slowed so that Ca^2+^ uptake by the SR decreased, this would cause cytosolic Ca^2+^ levels to increase and allow continuous formation of crossbridges. The fact that the baseline of our force traces returns to pre-stimulus levels when the test is over supports the idea that there are elevated levels of cytoplasmic Ca^2+^ that return to normal following stimulation. However, the time to return to baseline is prolonged (10–15 sec), so that depletion of ATP may also be involved which might cause prolonged binding of crossbridges. If the crossbridges are bound longer rather than cycling to produce this unrelaxed force, relatively little energy would be required between stimulus trains to retain a contracture-like state. Obviously, this phenomenon needs further study to elucidate the causal mechanisms, but its functional significance as a sexually dimorphic property may be to provide maintenance of relatively high forces for prolonged periods without fatigue (Peters and Aulner, 2000; Clark and Peters, 2006; Navas and James, 2007). Whether and to what extent it may save energy has yet to be determined.

In the T_+_ males, sustained force in both AIL and FCR reached levels (mean≈60% and 80%, respectively) a bit lower but still within the range of those found in normal males (mean≈65% and 85%, respectively) (Peters and Aulner, 2000). There was no difference in the amount of sustained force between T_+_ and T_0_ males for AIL, but FCR in the T_+_ males did reach a significantly greater level of sustained force at 2, 3 and 4 min than did the T_0_ males (Fig. 3). For FCR, the T_0_ values at two minutes (≈15% of total force) and three minutes (≈40%) into the fatigue test were intermediate between the normal males (≈37% and 60%, at 2 and 3 min) and females (≈3% and 20% at 2 and 3 min), but by four minutes had risen to levels comparable to the females (T_0_≈42%, females≈48% of total force) (Peters and Aulner, 2000).

Thus, sustained force in FCR of the T_0_ males was initially higher than in females, but at four minutes was at the same level. These results suggest that the mechanism which produces sustained force is activated in males in spite of the lack of circulating testosterone. AIL appears less sensitive to this lack, and retains all of its potential for developing sustained force in T_0_ males. FCR in T_0_ males retains significant levels of sustained force, as was true of normal females whose sustained force rose to produce over 45% of total force by four minutes (Peters and Aulner, 2000). So the lack of T in the T_0_ males resulted in somewhat lower levels of sustained force than in T_+_ and normal males, suggesting that in FCR, T levels do have an enhancing effect on this property. The more interesting question is why in the FCR, females have an equally great potential for producing sustained force in the absence of stimulation by T.

Testosterone stimulates muscle enlargement, but whether it acts through androgen receptors (AR) directly or if it is converted to estradiol (E2) and then binds estrogen receptors (ER) is unknown. In a study of orchidectomized mice treated with E2 and with non-aromatizable dihydrotestosterone (DHT) which can only act via androgen receptors, both hormones were found to increase muscle mass when compared to non-orchidectomized mice, but DHT maintained muscle mass to a greater degree (Svensson et al., 2010). In addition, Svensson et al. used microarray analysis to show that both DHT and E2 affect genes involved in regulation of muscle size (Svensson et al., 2010), but a greater number of these genes are regulated by DHT treatment in the muscles analyzed. Thus, it is likely that DHT upregulates a greater number of proteins involved in muscle preservation. In mice, ovariectomy decreases skeletal muscle mass and E2 supplementation restores it (Svensson et al., 2010). These results suggest that the steroid hormones bind these receptors in both males and females and either E2 or T can affect sustained force in both sexes. It is likely that male dimorphic muscles have more receptors than do females, enhancing sustained force. FCR may simply have more receptors than AIL in females, resulting in the greater sustained force in female FCRs.

Our data show more differences in the contractile properties of FCR between T_+_ and T_0_ males than in AIL, suggesting that FCR is more sensitive to the lack of T than is AIL. This may also result from differences in the numbers of AR receptors between the muscles and whether expression of these receptors decreases in the non-breeding season. Further studies examining the AR densities in breeding and non-breeding animals should provide better understanding of their role in the selective sexual dimorphism found in these muscles.

### Contractile properties and testosterone

The fact that few of the differences in individual comparisons in contractile properties reached significance (Table 1) may indicate that lack of T has a limited effect on reversing the masculinization of adult male muscles. Although, given the large variation in our results, a larger sample size may have produced more pair-wise differences. When comparing the mean tetanic force in AIL and FCR among normal, T_+_, T_0_ males and females (Fig. 5), the female values were consistently the lowest, as expected. The T_0_ males, though not significantly different from females tended to be intermediate between females and T_+_ or normal males. The CT and ½ RT pattern was similar, with the mean values for females lowest, T_0_ males tending to be intermediate, but not significantly lower than normal males or T_+_ males. So lack of T (at least for the 8–9 week period of this study) did not result in fully feminized properties in the T_0_ males.

The sexually dimorphic musculature experiences greater effects seemingly because they are more dependent on T for their unique properties, however, the intermediate nature of force production, contraction and relaxation times and fatigability of the T_0_ males compared with females suggests that there is an initial masculinizing effect of T which is heightened during breeding season, but not completely reversed outside breeding season. In mammals, T triggers development of masculine characters (e.g. laryngeal enlargement, lion's mane, larger body size), that do not require continuous high levels of T to maintain. Reduced T outside the breeding season in most species may be advantageous to the overall health of an animal because high T levels are known to depress the immune system as a result of a rise in glucocorticoids. The ability to preserve masculine characters that are triggered at puberty, independent of blood levels of T, may be adaptive in minimizing these effects while maintaining masculine properties and behaviors.

Our study of the contractile properties of the sexually dimorphic forelimb muscles suggests that in frogs masculinization of these properties is triggered by the initial increase in T at their first breeding season, and may not require high circulating levels of T to maintain. Although, at each breeding season when T levels rise, these male properties are indeed enhanced. In light of the use of these muscles in their competitive breeding behaviors, it is advantageous for males to respond rapidly to a seasonal rise in T and quickly achieve full breeding condition for grappling and amplexus. It may also cost less energy to simply maintain the elements necessary for muscle function at partially reduced levels, rather than build them anew each year.
